# Association of Common Variants in *LOX* with Keratoconus: A Meta-Analysis

**DOI:** 10.1371/journal.pone.0145815

**Published:** 2015-12-29

**Authors:** Jing Zhang, Lu Zhang, Jiaxu Hong, Dan Wu, Jianjiang Xu

**Affiliations:** 1 Research Center, Eye & ENT Hospital of Fudan University, Key Laboratory of myopia, Ministry of Health, Shanghai, China; 2 Department of Ophthalmology and Visual Science, Eye & ENT Hospital of Fudan University, Key Laboratory of myopia, Ministry of Health, Shanghai, China; 3 Department of Computer Science, City University of Hong Kong, Hong Kong, China; 4 Massachusetts Eye and Ear Infirmary, Harvard Medical School, Boston, Massachusetts, United States of America; Save Sight Institute, AUSTRALIA

## Abstract

**Background:**

Several case-control studies have been performed to examine the association of genetic variants in lysyl oxidase (*LOX*) with keratoconus. However, the results remained inconclusive and great heterogeneity might exist across populations.

**Method:**

A comprehensive literature search for studies that published up to June 25, 2015 was performed. Summary odds ratios (OR) and 95% confidence intervals (CI) of each single nucleotide polymorphism (SNP) were estimated with fixed effects model when *I* ^*2*^<50% in the test for heterogeneity or random effects model when *I* ^*2*^>50%. Publication bias was evaluated using funnel plots and Egger’s test.

**Results:**

A total of four studies including 1,467 keratoconus cases and 4,490 controls were involved in this meta-analysis. SNPs rs2956540 and rs10519694 showed significant association with keratoconus, with ORs of 0.71 (95% CI: 0.63–0.80, *P* = 1.43E-08) and 0.77 (95% CI: 0.61–0.97, *P* = 0.026), respectively. In contrast, our study lacked sufficient evidences to support the association of rs1800449/rs2288393 with keratoconus across populations.

**Conclusion:**

This meta-analysis suggested that two *LOX* variants, rs2956540 and rs10519694, may affect individual susceptibility to keratoconus, while distinct heterogeneity existed within this locus. Larger-scale and multi-ethnic genetic studies on keratoconus are required to further validate the results.

## Introduction

Keratoconus is a bilateral, non-inflammatory corneal ectasia characterized by progressive thinning and conical protrusion of the cornea, which eventually leads to mild or markedly impaired vision due to high myopia and irregular astigmatism. The approximated incidence of keratoconus is 1 in 2,000 individuals in the general population and the estimated prevalence is reported to be 54.5 per 100,000 [1]. Previous studies suggested that the incidence rates and prevalence are much higher in Asians than Caucasians, implying substantial effects of population differences underlying this disease [2–4]. Owing to the limited availability of medical treatments, end-stage keratoconus patients have no other choice but to accept corneal transplantation, while repeated operations might be necessary following rejection or failure of a prior corneal transplant. Therefore, it will be of importance and desirable if keratoconus could be diagnosed at earlier stages.

The etiology of keratoconus is far from understood, with environmental, behavioral, and genetic factors all contributing to the disease. However, the much higher risk of keratoconus in first-degree relatives compared with the general population, and the high disease concordance in monozygotic twins, indicate a strong genetic component to keratoconus [5–7]. While the genetic etiology of many other corneal dystrophies has been identified, the understanding of the genetic background of keratoconus has proven more complex. Many keratoconus loci have been identified by genetic linkage analysis, whilst few of them have been replicated independently [8]. Specific candidate genes related to these loci have been repeatedly investigated by mutational screening, including *VSX1*[9], *SOD1*[10], *TGFBI*[11], and multiple collagen genes (*COL4A1-4*, *COL5A1*)[12, 13]. However, these genes explained only a small proportion of heritability in sporadic keratoconus cases [7], and the results were somewhat complicated, with some pedigrees showing associations with mutations in these genes and others were not [6].

Genome-wide association studies (GWAS) have also been performed in keratoconus, albeit in relatively small number of samples. In 2012 a GWAS project consisting of 526 keratoconus cases and 3,842 controls, together with 70 keratoconus families with a total of 146 patients and 161 unaffected family members identified lysyl oxidase (*LOX*) as a potential susceptibility gene for keratoconus in American Caucasians [14]. SNPs rs10519694 and rs2956540 that located in intron 4 of *LOX* showed strong association with the disease in that GWAS study, although their overall *P*-values did not reach genome-wide significance in the combined dataset. SNPs rs1800449 and rs2288393, which are in absolute linkage disequilibrium (LD, *r* ^*2*^ = 1 in Caucasians, CEU), revealed marginal association with the disease in case/control cohort from the same study. Later, replication studies in Czech Caucasians [15] and in Chinese [16] only supported the association of rs2956540 with keratoconus. Other SNP like rs1800449 was reported to be associated with keratoconus in an Iranian population [17]. Although this evidence highlighted the potential contribution of *LOX* to keratoconus susceptibility, the results were inconclusive and great heterogeneity might exist across populations. Moreover, individual study might be restricted by sample size and other limitations of study design, while meta-analysis might benefit in overcoming the limitations by increasing the sample size, which has been widely applied in genetic association studies. Therefore, the aim of the current study was designed to clarify the association between *LOX* variants and keratoconus susceptibility.

## Materials and Methods

### Identification of eligible studies

All studies investigating the association of variants in *LOX* with keratoconus were fully considered and carefully selected. Data were extracted from the following electronic databases: PubMed, Google scholar, Chinese National Knowledge Infrastructure (CNKI), and Weipu database (accessed June 25, 2015), with the following MeSH terms and free words: “*LOX*”, “lysyl oxidase”, “polymorphism”, “SNP”, “variant(s)”, “single nucleotide polymorphism” and “keratoconus”. Studies published in English and Chinese were all included in this meta-analysis, and the reference lists of all the relevant studies, including original articles, reviews were carefully screened. The inclusion criteria applied in the review process was shown as following: (1) case-control studies that investigating the association of *LOX* variants with keratoconus. Familial studies were excluded due to the relationships between individuals biasing the allele counting analysis methods employed for meta-analysis; (2) subjects from case and control groups should come from the same geographically and temporally defined population, and control subjects were free of keratoconus or any form of ocular diseases; (3) Sufficient data for estimating odds ratio (OR) with 95% confidence interval (CI). Data from non-overlapped sample cohorts from the same study were considered as different sample collections as introduced previously [18].

### Data extraction

Two reviewers independently assessed all potentially relevant studies with the inclusion criteria, cross-checked, discussed all conflict, and reached consensus on all items. The following information was extracted from each study: author, year of publication, population ethnicity, age and gender information of cases and controls, the result of Hardy-Weinberg equilibrium (HWE) test in controls, numbers of cases and controls, and available allele and/or genotype counts or frequencies of each SNP. The allele or genotype counts were calculated from the frequencies, rounding to the closest integer, in those studies where the counts were not presented [14–16].

### Meta-analysis and evaluation of publication bias

SNPs reported in two or more studies were conducted for meta-analysis. The heterogeneity among studies was tested with the *I* ^*2*^ test. OR and 95% CI for the minor allele were calculated with fixed effects model when *I* ^*2*^<50% or random effects model when *I* ^*2*^>50% [19]. The statistical significance of the association between SNPs in *LOX* and keratoconus was evaluated by the *Z*-test. The *P* values were transformed from the Z scores and a pooled *P* value less than 0.05 was considered as statistically significant. Publication bias was evaluated using a funnel plot, in which the standard error of log (OR) of each study was plotted against its OR. An asymmetric plot suggested possible publication bias. Funnel plot asymmetry was further evaluated by the method of Egger’s test [20] or Horbold-Egger’s test [21]. When the Egger’s test or Horbold-Egger’s test reported *P*<0.05, publication bias was assumed to exist. The LD values between SNPs in different populations were extracted from SNAP website based on the data from 1000 Genome Project[22] (https://www.broadinstitute.org/mpg/snap/).

## Results

### Literature search and characteristics of eligible studies

**Fig 1:**
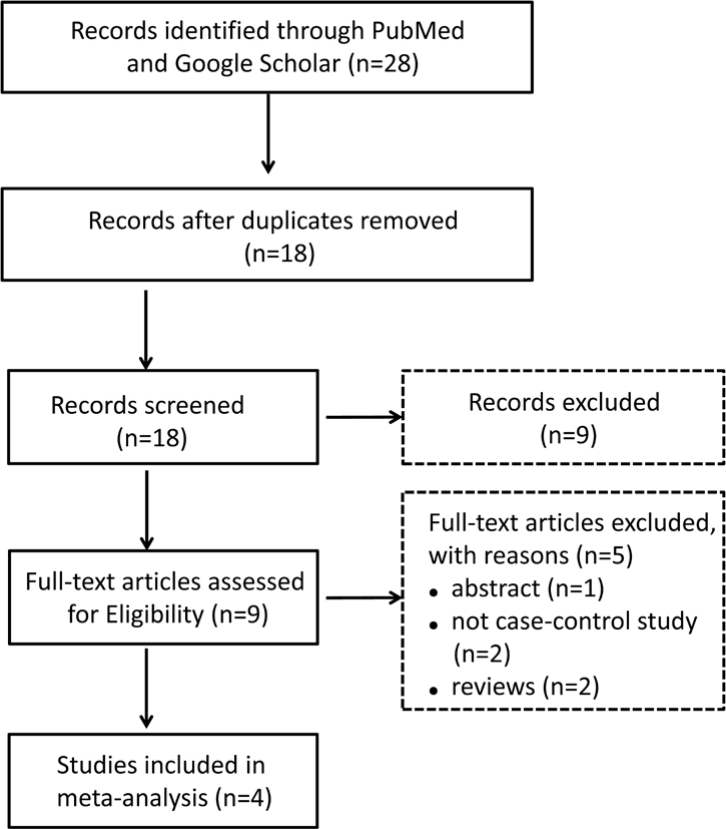
Flow chart of literature search and study selection.

Fig 1 outlined our study selection process. Briefly, a total of 28 articles including 18 independent studies were identified after an initial search from the selected electronic databases. Nine studies were excluded since they focused on the biological activity of *LOX* rather than its genetic contribution to keratoconus. Two studies were excluded since they were not case-control studies, and additional three studies with not enough available data were further excluded (S2 Appendix). Finally, four articles involving one GWAS [14] and three replication studies [15–17] were included in the meta-analysis (S1 Appendix). Three hundred and seven individuals from the study of Bykhovskaya *et al*. were excluded since they were from keratoconus families. Thus, a total of 1,467 keratoconus cases and 4,490 controls from six sample collections were included. The basic characteristics of all the included articles were summarized in Table 1.

**Table 1:** Characteristics of the studies included in the meta-analysis. HWE: Hardy-Weinberg equilibrium.

Study	Country	Sample size		Gender(%Female)		Age, mean±SD,years		HWE
		Cases	Controls	Cases	Controls	Cases	Controls	
Bykhovskaya 2012a [14]	American Caucasians	222	3324	NA	NA	NA	NA	YES
Bykhovskaya 2012b [14]	American Caucasians	304	518	NA	NA	NA	NA	YES
Bykhovskaya 2012c [14]	American Caucasians	377	191	NA	NA	NA	NA	YES
Hasanian-Langroudi 2014 [17]	Iranian	112	150	50%	56%	29.66±13.4	29.87±15.6	YES
Dudakova 2015 [15]	Czech Caucasians	165	193	34.5%	40.9%	37.2±13.3	39.5±13.7	NA
Hao 2015 [16]	Chinese	210	191	14.4%	24.2%	21.0±5.6	26.8±11.5	YES

### Meta-analysis of variants in LOX and keratoconus

**Fig 2:**
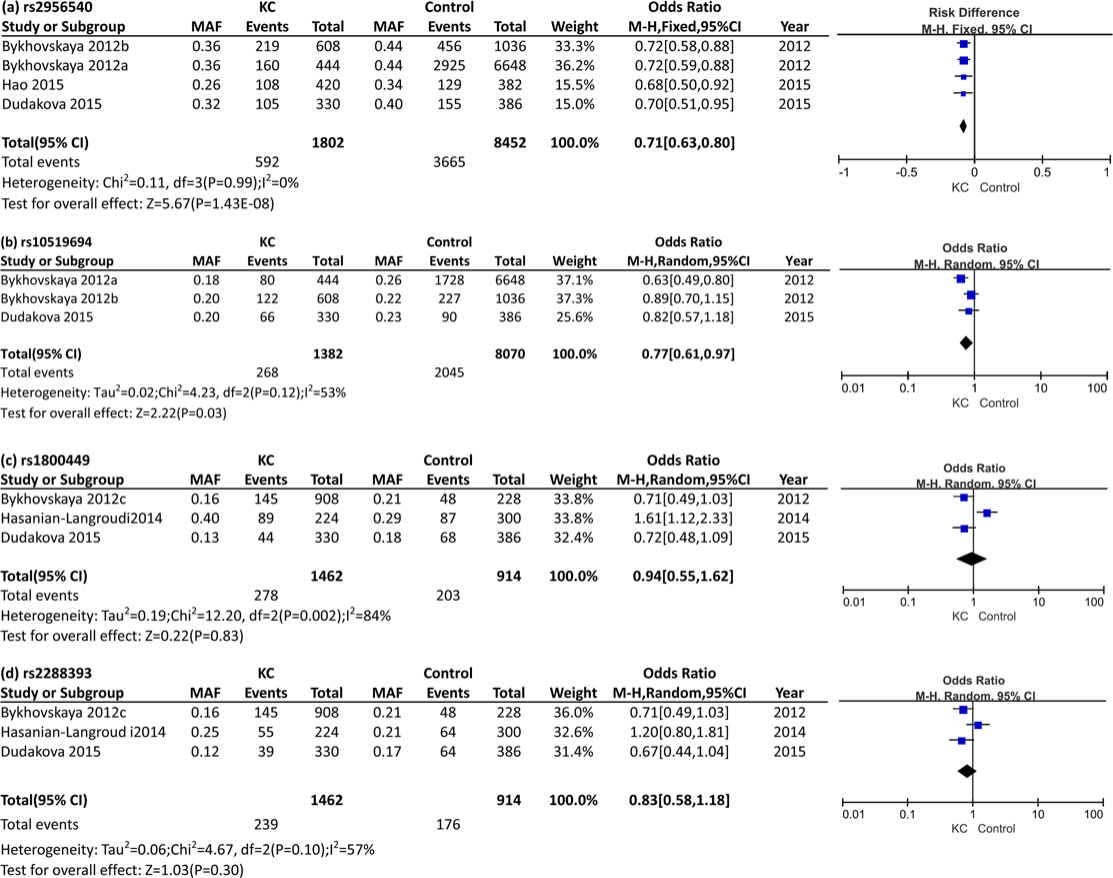
Forest plot of *LOX* allelic model: (a) *LOX* rs2956540; (b) *LOX* rs10519694; (c) *LOX* rs1800449; (d) *LOX* rs2288393. Events indicated the minor allele count of each SNP; MAF indicated the minor allele frequency. Squares indicated the study-specific odds ratio (OR). The size of the box was proportional to the weight of the study. Horizontal lines showed 95% confidence interval (CI). A diamond indicated the summary OR with its corresponding 95% CI.

Four SNPs (rs2956540, rs10519694, rs1800449, and rs2288393) in *LOX* were performed for meta-analysis. For SNP rs2956540, there were three studies from Caucasian populations and one study from Asian population, including a total of 901 keratoconus cases and 4226 controls. No obvious between-study heterogeneity was observed and thus the fixed-effects model was applied. The C allele of rs2956540 was significantly associated with keratoconus, conferring a pooled OR of 0.71 (95% CI: 0.63–0.80, *P* = 1.43E-08; Fig 2A and Table 2). SNP rs10519694 was investigated in Caucasian populations only, while moderate heterogeneity (*I* ^*2*^ = 53%) was detected, which might be explained by the differences between Czech Caucasians and American Caucasians. The random effects model was adopted here and this SNP was also significantly associated with keratoconus (OR = 0.77, 95% CI: 0.61–0.97, *P* = 0.026; Fig 2B and Table 2). SNP rs1800449 and rs2288393, which are in absolute LD with each other in American Caucasians, showed opposite trends among the involved three studies. The pooled ORs for the two SNPs were not statistically significant, and distinct heterogeneity was detected (Fig 2C and 2D and Table 2), which could be explained by the population differences for the two SNPs between Iranian cohort and the other two Caucasian cohorts. When the data from the Iranian cohort was not considered, the association of the two SNPs in Caucasians became significant (rs1800449: OR = 0.72, 95% CI: 0.54–0.94, *P* = 0.02, *I* ^*2*^ = 0%; rs2288393: OR = 0.70, 95% CI: 0.53–0.92, *P* = 0.01, *I* ^*2*^ = 0%). Since the genotype counts/frequency data was not available in the studies of Bykhovskaya *et al*. and Dudakova *et al*., meta-analyses on the genotypic level were not performed in the current study.

**Table 2:** Meta-analysis of allelic association of LOX polymorphisms with keratoconus. The ORs (odds ratios) were calculated with respect to the minor allele of each SNP, which were shown in bold font in Table 2.

Polymorphism	Alleles	Number of study cohorts	sample size (case/control)	OR (95% CI)	Z score	*P*-values	*I* ^*2*^ (%)	Model
rs2956540	G>**C**	4	901/4226	0.71 (0.63–0.80)	5.67	1.43E-08	0	Fixed effect
rs10519694	C>**T**	3	691/4035	0.76 (0.65–0.88)	2.22	0.026	53	Random effect
rs1800449	C>**T**	3	731/457	0.94 (0.55–1.62)	0.22	0.83	84	Random effect
		2#	619/307	0.72 (0.54–0.94)	2.4	0.02	0	Fixed effect
rs2288393*	C>**G**	3	731/457	0.83 (0.58–1.18)	1.03	0.30	57	Random effect
		2#	619/307	0.70 (0.53–0.92)	2.57	0.011	0	Fixed effect

* The MAF of rs1800449 in Bykhovskaya 2012c was used here, due to their demonstrated absolute LD.

# Caucasians only

### Test for potential biases

Publication bias were qualitatively assessed by funnel plot and quantitatively assessed by Egger’s test or Horbold-Egger’s test. None of the test detected obvious evidence of publication bias (S1 Fig).

## Discussion

In a recent genome-wide association study, Bykhovskaya *et al*. suggested that two SNPs in *LOX* gene, rs2956540 and rs10519694 contributed to keratoconus susceptibility, although their overall *P* values did not reach genome-wide significance. After sequencing of *LOX* gene in affected individuals and testing of transcribed *LOX* polymorphisms in relatively small case-control and family cohorts, they further indicated the additional marginal effect of rs1800449/rs2288393 to the overall association signal in the *LOX* gene. These SNPs have been subsequently investigated in different ethnic populations whilst the results were inconsistent and variable. Therefore, in order to provide a reliable evaluation of the association between SNPs in *LOX* and keratoconus susceptibility, we performed meta-analysis, which involved the largest sample size to date with a total of 1,467 keratoconus cases and 4,490 controls. A strong association of *LOX* variant rs2956540 with keratoconus was identified in the combined dataset of Caucasians and Chinese cohorts (*P* = 1.43E-08) and there was no heterogeneity (*I* ^*2*^ = 0). Our data also suggested the significant association of rs10519694 with keratoconus (*P* = 0.026) in Caucasians, with moderate heterogeneity (*I* ^*2*^ = 53%) probably due to the underlying population differences. This SNP has not been replicated in other ethnicities, and its contribution to keratoconus susceptibility in non-Caucasians requires further investigation. In contrast, our study lacked sufficient evidences to support the association of rs1800449/rs2288393 with keratoconus (*P*>0.01) across populations. Distinct heterogeneity has been observed across study populations (*I* ^*2*^>50%).

To our knowledge, this is the first meta-analysis report evaluating the published genetic studies on *LOX* in keratoconus, and this study identified a keratoconus-associated SNP that first reaching genome-wide significance at this locus. *LOX* encodes an extracellular copper enzyme that initiates the crosslinking of collagens and elastin. The formation of covalent bonds between collagen and elastin fibrils, which maintain the biomechanical properties of the cornea, is mediated by LOX and other four lysyl oxidase-like enzymes. It was demonstrated that changes in LOX distribution and its decreased activity might be potential reasons for the inadequate collagen cross-linking in keratoconus, which is a hallmark of this disease [23]. In addition, a significant reduction in *LOX* transcript was observed in corneal epithelia of keratoconus patients compared to healthy donors, and LOX activity in keratoconus tears was found to correlate with disease severity [24]. All of these evidences suggested the critical involvement of *LOX* in the pathogenesis of keratoconus, and also implying the importance of deciphering the genetic code of *LOX* in contributing keratoconus susceptibility. Rs2956540, the most significant SNP at this locus showed remarkable consistency of disease association across populations, although its minor allele frequency was quite different among populations (S1 Table). SNP rs2956540 may affect gene expression through transcriptional regulation, as predicted by Genomatrix (http://www.genomatix.de/) that it can alter the binding sites of several transcription factors like PTX1, CMYB, and ISM1. Our study highlighted the importance of *LOX* locus to keratoconus susceptibility, while more efforts are required to get a comprehensive understanding on the detailed mechanisms.

Heterogeneity is a problem that might influence the results of our meta-analysis. Except for rs2956540, which showed highly consistent effects among the overall population analysis, significant heterogeneity was observed for the other three SNPs. The genetic effects of rs1800449 and rs2288393 in Iranian cohort were even opposite to that of in Caucasian populations. The four interrogated SNPs showed different allele frequency in the various populations (shown in S1 Table). We also examined the correlation of these SNPs based on the data from 1000 Genome Project, and found the LD patterns of the four investigated SNPs were quite different among Caucasians (CEU), Asians (Chinese + Japanese, CHB+JPT), and Yoruba in Ibadan, Nigeria (YRI). The *r* ^*2*^ values of these SNPs in different ethnicities were shown in S2 Table. The heterogeneity might arise from patients’ genetic backgrounds, like different allele frequency and LD patterns of these SNPs among populations, or differences in average age, lifestyles, or subclinical forms of the disease and differences in diagnostic methods and criteria. However, due to the limited number of studies that evaluating the association of *LOX* variants to keratoconus susceptibility, currently we cannot perform further stratified analysis based on ethnicity, subclinical forms, or sample size. In addition, the contributions of these variants to keratoconus susceptibility were investigated in only a few populations, it should be of higher importance and generalizability if more details and various ethnicities in relation to keratoconus were reported, especially in Asians.

Several other limitations of this meta-analysis should also be noted. Firstly, this meta-analysis was performed based on studies published in English and Chinese. It is possible that some relevant articles written by other languages may be missed out, which might introduce selection and publication bias, although we did not detect any significant bias by Egger’s test in the current study. Secondly, we failed to obtain original data from the studies included, making it unlikely to conduct a more precise analysis based on the adjustment of age, gender, or some other factors. Thirdly, only a small number of studies were eligible for inclusion in this meta-analysis. Although over 1,400 cases were involved in total, no single analysis included this many patients. Thus, the conclusions must be validated by further studies, and it should also be updated by including more upcoming reports.

In conclusion, the current study provided substantial evidences that two *LOX* variants, rs2956540 and rs10519694 were significantly associated with keratoconus. In the future, the two identified SNPs may be included in a genetic risk score with other associated SNPs for disease risk prediction. Future large-scale and multi-ethnic genetic studies on keratoconus are warranted, thereby ensuring a more comprehensive understanding of the genetic basis of keratoconus.

## Supporting Information

S1 Checklist Checklist for meta-analysis on genetic association studies. (DOCX)

S1 Appendix Lists of included studies. (DOCX)

S2 Appendix Lists of excluded studies with reasons. (DOCX)

S1 Fig Publication bias analyses. (DOCX)

S1 Table Minor allele frequency of the four investigated SNPs in *LOX* gene from different populations (Shown were data from 1000 Genome project pilot 1). (DOCX)

S2 Table LD pattern of the four investigated SNPs in *LOX* gene from different populations (Shown were *r* ^*2*^ values from 1000 Genome project). (DOCX)
