# Supplementary material for: Association of Common Variants in LOX with Keratoconus: A Meta-Analysis
Source: PLoS One. 2015 Dec 29;10(12):e0145815. doi: 10.1371/journal.pone.0145815 (PMC4699887; doi:10.1371/journal.pone.0145815)
Supplement: S1 Appendix — (DOCX) [file pone.0145815.s002.docx]

Appendix S1 Lists of included studies

[[1-4](#_ENREF_1)]

1. Dudakova, L., et al., *Validation of rs2956540:G>C and rs3735520:G>A association with keratoconus in a population of European descent.* Eur J Hum Genet, 2015.

2. Hao, X.D., et al., *Evaluating the Association between Keratoconus and Reported Genetic Loci in a Han Chinese Population.* Ophthalmic Genet, 2015. **36**(2): p. 132-6.

3. Hasanian-Langroudi, F., et al., *Association of Lysyl oxidase (LOX) polymorphisms with the risk of Keratoconus in an Iranian population.* Ophthalmic Genet, 2014.

4. Bykhovskaya, Y., et al., *Variation in the lysyl oxidase (LOX) gene is associated with keratoconus in family-based and case-control studies.* Invest Ophthalmol Vis Sci, 2012. **53**(7): p. 4152-7.
