# Supplementary material for: Association of Common Variants in LOX with Keratoconus: A Meta-Analysis
Source: PLoS One. 2015 Dec 29;10(12):e0145815. doi: 10.1371/journal.pone.0145815 (PMC4699887; doi:10.1371/journal.pone.0145815)
Supplement: S2 Appendix — (DOCX) [file pone.0145815.s003.docx]

Appendix S2 Lists of excluded studies with reason

Article [[1](#_ENREF_1)] studied gene expression pattern rather than genetic polymorphisms in keratoconus subjects.

Article [[2](#_ENREF_2)] studied the biological activity of LOX but not polymorphisms. .

Article [[3](#_ENREF_3)] studied the biological activity of LOX but not polymorphisms.

Article [[4](#_ENREF_4)] focused on the biological activity of LOX but not polymorphisms.

Article [[5](#_ENREF_5)] focused on the biological activity of LOX but not polymorphisms.

Article [[6](#_ENREF_6)] focused on the biological activity of LOX but not polymorphisms.

Article [[7](#_ENREF_7)] focused on the biological activity of LOX but not polymorphisms.

Article [[8](#_ENREF_8)] was a review and excluded due to its nature.

Article [[9](#_ENREF_9)] was a review and excluded due to its nature.

Article [[10](#_ENREF_10)] was a review and excluded due to its nature.

Article [[11](#_ENREF_11)] performed linkage-analysis rather than case-control association study in keratoconus.

Article [[12](#_ENREF_12)] performed mutation screening on keratoconus cases only.

Paper [13] was an abstract of a poster and data are insufficient for analysis.

Paper [14] was an abstract of a poster and data are insufficient for analysis.

1. Nielsen, K., et al., *Identification of differentially expressed genes in keratoconus epithelium analyzed on microarrays.* Invest Ophthalmol Vis Sci, 2003. **44**(6): p. 2466-76.

2. Avetisov, S.E., V.R. Mamikonian, and I.A. Novikov, *[The role of tear acidity and Cu-cofactor of lysyl oxidase activity in the pathogenesis of keratoconus].* Vestn Oftalmol, 2011. **127**(2): p. 3-8.

3. Dudakova, L., et al., *Changes in lysyl oxidase (LOX) distribution and its decreased activity in keratoconus corneas.* Exp Eye Res, 2012. **104**: p. 74-81.

4. Sethi, A., R.J. Wordinger, and A.F. Clark, *Focus on molecules: lysyl oxidase.* Exp Eye Res, 2012. **104**: p. 97-8.

5. Dudakova, L. and K. Jirsova, *The impairment of lysyl oxidase in keratoconus and in keratoconus-associated disorders.* J Neural Transm, 2013. **120**(6): p. 977-82.

6. Shetty, R., et al., *Attenuation of lysyl oxidase and collagen gene expression in keratoconus patient corneal epithelium corresponds to disease severity.* Mol Vis, 2015. **21**: p. 12-25.

7. Wojcik, K.A., et al., *Role of biochemical factors in the pathogenesis of keratoconus.* Acta Biochim Pol, 2014. **61**(1): p. 55-62.

8. Dudakova, L., P. Liskova, and K. Jirsova, *Is copper imbalance an environmental factor influencing keratoconus development?* Med Hypotheses, 2015. **84**(5): p. 518-24.

9. Jeyabalan, N., et al., *Genetic and genomic perspective to understand the molecular pathogenesis of keratoconus.* Indian J Ophthalmol, 2013. **61**(8): p. 384-8.

10. Burdon, K.P. and A.L. Vincent, *Insights into keratoconus from a genetic perspective.* Clin Exp Optom, 2013. **96**(2): p. 146-54.

11. Bisceglia, L., et al., *Linkage analysis in keratoconus: replication of locus 5q21.2 and identification of other suggestive Loci.* Invest Ophthalmol Vis Sci, 2009. **50**(3): p. 1081-6.

12. De Bonis, P., et al., *Mutational screening of VSX1, SPARC, SOD1, LOX, and TIMP3 in keratoconus.* Mol Vis, 2011. **17**: p. 2482-94.

13. Bykhovskaya, Y., Rasheed, A., & Rabinowitz, Y. S. *LOX gene polymorphisms are associated with keratoconus and predicted to affect protein structure.* Investigative Ophthalmology & Visual Science, 2015. **56**(7), p.2042-2042.

14. Dudakova, L., Kalasova, S., & Jirsova, K. *Presence of lysyl oxidase-like enzymes in human control and keratoconic corneas.* Investigative Ophthalmology & Visual Science, 2013.**54**(15), p.5293-5293.
