# Supplementary material for: Association of Common Variants in LOX with Keratoconus: A Meta-Analysis
Source: PLoS One. 2015 Dec 29;10(12):e0145815. doi: 10.1371/journal.pone.0145815 (PMC4699887; doi:10.1371/journal.pone.0145815)

**Supplementary Figure 1. Publication bias analyses.**

Publication bias was evaluated using a funnel plot, in which the standard error of log(OR) of each study was plotted against its OR. Funnel plot asymmetry was further evaluated by the method of Egger’s test or Horbold-Egger’s test. When the Egger’s test or Horbold-Egger’s test reported *P*<0.05, publication bias was assumed to exist.

1. rs2956540: Egger’s test *P*=0.0616


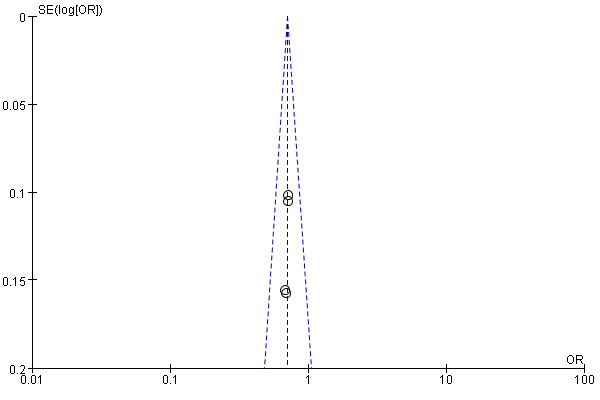


1. rs10519694: Horbold-Egger’s test *P*=0.7069


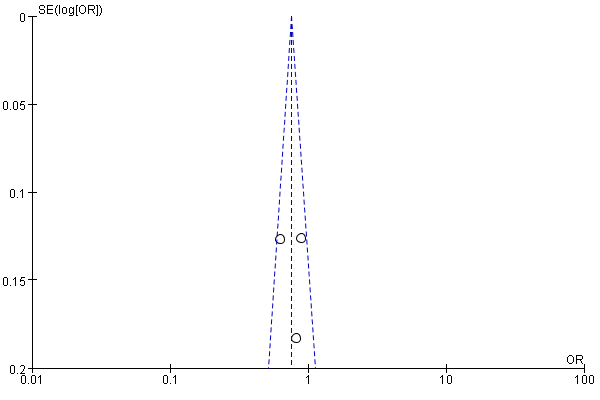


(c) rs1800449: Horbold-Egger’s test *P*=0.325


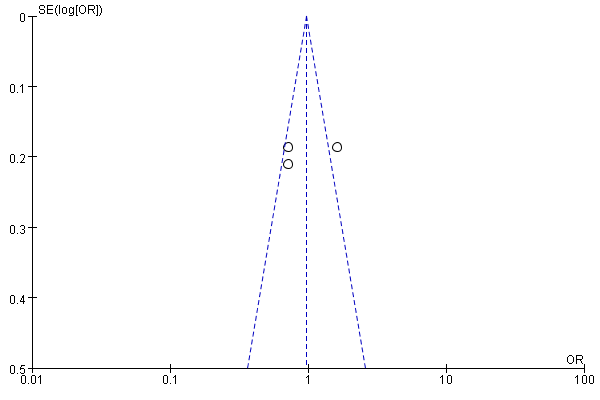


(d) rs2288393: Horbold-Egger’s test *P*=0.776


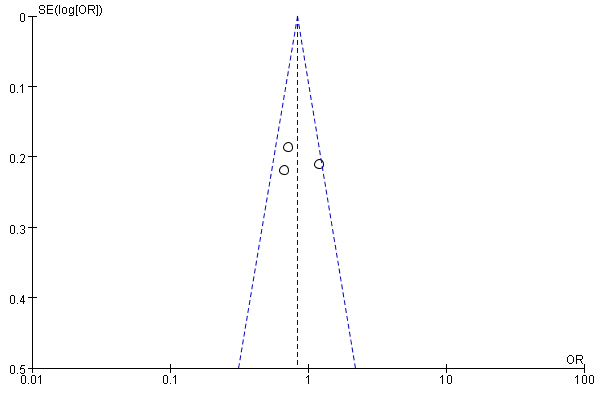

Supplement: S1 Fig — (DOCX) [file pone.0145815.s004.docx]
