# Supplementary material for: Association of Common Variants in LOX with Keratoconus: A Meta-Analysis
Source: PLoS One. 2015 Dec 29;10(12):e0145815. doi: 10.1371/journal.pone.0145815 (PMC4699887; doi:10.1371/journal.pone.0145815)
Supplement: S1 Table — (DOCX) [file pone.0145815.s005.docx]

**Supplementary Table 1.** Minor allele frequency of the four investigated SNPs in *LOX* gene from different populations (Shown were data from 1000 Genome project pilot 1)

|  | rs2956540 (G) | rs10519694 (T) | rs1800449 (T) | rs2288393 (C) |
| --- | --- | --- | --- | --- |
| YRI | 0.195 | 0.017 | 0.136 | 0.042 |
| CEU | 0.383 | 0.275 | 0.108 | 0.108 |
| CHB+JPT | 0.225 | 0.042 | 0.175 | 0.142 |

YRI: Yoruba in Ibadan, Nigeria; CEU: Caucasians; CHB: Chinese; JPT: Japanese.
