# Supplementary material for: Association of Common Variants in LOX with Keratoconus: A Meta-Analysis
Source: PLoS One. 2015 Dec 29;10(12):e0145815. doi: 10.1371/journal.pone.0145815 (PMC4699887; doi:10.1371/journal.pone.0145815)
Supplement: S2 Table — (DOCX) [file pone.0145815.s006.docx]

**Supplementary Table 2.** LD pattern of the four investigated SNPs in *LOX* gene from different populations (Shown were *r^2^* values from 1000 Genome project)

| **CEU** | rs2956540 | rs10519694 | rs1800449 | rs2288393 |
| --- | --- | --- | --- | --- |
| rs2956540 | / | 0.61 | 0.195 | 0.195 |
| rs10519694 | / | / | 0.046 | 0.046 |
| rs1800449 | / | / | / | 1 |
| rs2288393 | / | / | / | / |

| **CHB+JPT** | rs2956540 | rs10519694 | rs1800449 | rs2288393 |
| --- | --- | --- | --- | --- |
| rs2956540 | / | 0.15 | 0.731 | 0.569 |
| rs10519694 | / | / | 0.001 | 0.004 |
| rs1800449 | / | / | / | 0.778 |
| rs2288393 | / | / | / | / |

| **YRI** | rs2956540 | rs10519694 | rs1800449 | rs2288393 |
| --- | --- | --- | --- | --- |
| rs2956540 | / | 0.004 | 0.143 | 0.183 |
| rs10519694 | / | / | 0.003 | 0.001 |
| rs1800449 | / | / | / | 0.007 |
| rs2288393 | / | / | / | / |
